# Improvement in Salt Tolerance Ability of *Pseudomonas putida* KT2440

**DOI:** 10.3390/biology13060404

**Published:** 2024-06-01

**Authors:** Min Fan, Shuyu Tan, Wei Wang, Xuehong Zhang

**Affiliations:** State Key Laboratory of Microbial Metabolism, School of Life Sciences and Biotechnology, Shanghai Jiao Tong University, Shanghai 200240, China; fanmin8@sjtu.edu.cn (M.F.); tanshuyu@sjtu.edu.cn (S.T.); weiwang100@sjtu.edu.cn (W.W.)

**Keywords:** salt tolerance, *Pseudomonas putida*, bioremediation, synthetic biology

## Abstract

**Simple Summary:**

*Pseudomonas putida* KT2440 is an attractive platform for bioremediation with versatile growth substrate and tolerance capabilities. The limited research on the salt tolerance of strain KT2440 hinders its application in bioremediation in high-salinity environments, where the composition of pollutants becomes increasingly complex and wastewater salinity often remains high. Therefore, the salt tolerance of strain KT2440 was explored in this work. By analyzing transcriptomic data from the high-salinity minimal salts medium, we identified key salt-tolerance genes and used them to improve the salt tolerance of strain KT2440 under 4% *w*/*v* NaCl. With co-expression of salt-tolerant genes, the maximum salt tolerance of strain KT2440 increased to 5% *w*/*v* NaCl. Further addition of compatible solutes increased the salt tolerance of the strain to 6% *w*/*v* NaCl. The engineered strain KT2440 could degrade common pollutants under 4% *w*/*v* NaCl in 48 h, and could be a promising platform for bioremediation in high-salinity environments.

**Abstract:**

*Pseudomonas putida* KT2440 is a popular platform for bioremediation due to its robust tolerance to environmental stress and strong biodegradation capacity. Limited research on the salt tolerance of *P. putida* KT2440 has hindered its application. In this study, the strain KT2440 was tested to tolerate a maximum of 4% *w*/*v* NaCl cultured with minimal salts medium. Transcriptomic data in a high-salinity environment showed significant expression changes in genes in membrane components, redox processes, chemotaxis, and cellular catabolic processes. *betB*-encoding betaine-aldehyde dehydrogenase was identified from the transcriptome data to overexpress and enhance growth profile of the strain KT2440 in minimal salts medium containing 4% *w*/*v* NaCl. Meanwhile, screening for exogenous salt-tolerant genes revealed that the Na^+^/H^+^ antiporter *EcnhaA* from *Escherichia coli* significantly increased the growth of the strain KT2440 in 4% *w*/*v* NaCl. Then, co-expression of *EcnhaA* and *betB* (KT2440-*EcnhaA-betB*) increased the maximum salt tolerance of strain KT2440 to 5% *w*/*v* NaCl. Further addition of betaine and proline improved the salt tolerance of the engineered strain to 6% *w*/*v* NaCl. Finally, the engineered strain KT2440-*EcnhaA-betB* was able to degrade 56.70% of benzoic acid and 95.64% of protocatechuic acid in minimal salt medium containing 4% *w*/*v* NaCl in 48 h, while no biodegradation was observed in the normal strain KT2440 in the same conditions. However, the strain KT2440-*EcnhaA-betB* failed to degrade catechol in minimal salt medium containing 3% *w*/*v* NaCl. This study illustrated the improvement in the salt tolerance performance of *Pseudomonas putida* KT2440 and the feasibility of engineered strain KT2440 as a potential salt-tolerant bioremediation platform.

## 1. Introduction

Salt ions are essential for life. A low-salt environment causes cells to swell, rupture or even die, while a high-salt environment causes cells to lose water and die. On the one hand, salt ions provide the osmotic pressure to maintain cellular homeostasis; on the other hand, salt ions participate in biological processes as coenzymes. For microbes, they are classified into halotolerant and halophilic microorganisms dependent on their demand for salt [1]. Microorganisms utilize two primary strategies, a salt-out strategy and a salt-in strategy, to regulate osmolality to adapt to high-salt environments [2,3,4,5,6,7,8]. The salt-tolerant microorganisms and most halophilic microorganisms employ a salt-out strategy, which involves transporting salt ions to extracelluar conditions by functional membrane transporters and synthesizing or accumulating higher concentrations of compatible solutes to adaption to high-salinity environments. These transporters consist of two major groups: the primary sodium pump, including Na^+^-translocating decarboxylase [9,10,11], methyltetrahydromethanopterin:coenzyme M methyltransferase [12], vacuolar-type Na^+^-translocating ATPase [13] and Na^+^-translocating NADH:ubiquinone oxidoreductase [14,15]; and the Na^+^/H^+^ antiporters—NhaA family [16,17], NhaB family [18], NhaC family [19], NhaD family [20,21], etc. [22,23,24]. Microorganisms synthesize various compatible solutes to survive in hypersaline environments—glycine-betaine [25,26], proline [27,28], mannitol [29], trehalose [30], ectoine [31,32], etc. [25]. On the other hand, certain microorganisms such as *Halobacteriaceae* and *Salinibacte* harness the salt-in strategy, accumulating high intracellular concentrations of inorganic salts (KCl or NaCl) to maintain osmotic balance [33]. Therefore, these two strategies are commonly used to improve the salt tolerance of microbes. For instance, the heterologous expression of the Nha2 Na^+^/H^+^ antiporter from *Yarrowia lipolytica* or the K^+^ transporter AlHKT2;1 from *Aeluropus lagopoides* can improve the Na^+^ tolerance of *Saccharomyces cerevisiae* [34,35].

Environmental pollution caused by hazardous materials poses a major threat to human health and natural ecosystems. Bioremediation is the use of biological metabolic activities to reduce or mineralize toxic and harmful substances in the polluted environment. Because it is of low and environmentally friendly, microbial remediation has an important role in heavy metal removal and toxic substance degradation. For example, *Helianthus tuberosus* is capable of biosorption of heavy metal (e.g., Mn Zn and Ni) [36]. *Vicia faba* is capable of biosorption of cadmium [37]. Different microorganisms including bacteria (*Pseudomonas*, *Alcaligenes*, *Bacillus*, *Rhodococcus*, etc.), fungi (*Aspergillus*, *Penecillium*, etc.), and yeasts (*Pichia*, *Rhodotorula*, etc.) have been reported to be involved in the efficient biodegradation of pollutants from contaminated environments, due to their exceptional bioremediation ability [38]. However, various environmental conditions (e.g., pH, temperature and salinity) of the contaminated site may affect the performance of the microbial remediation, in particular in the high-salinity environment. Currently, fewer studies have been reported to improve salt tolerance traits in environmental microorganisms. Therefore, it is necessary to improve the salt tolerance of environmental microorganisms for bioremediation.

*Pseudomonas putida* KT2440, an ideal chassis for industrial biotechnology and bioremediation, has attracted increasing interest from researchers [39,40,41,42]. Recently, researchers make great efforts to reveal and elucidate the functional gene for tolerance under various stress conditions of *P. putida* KT2440 and to expand its biotechnological applications, such as a major facilitator superfamily transporter involved in propionic acid tolerance [43], an ABC transporters involved in furfural tolerance [44], and the efflux pump protein TtgB involved in *p*-coumaric and ferulic acid tolerance [45]. Some researchers engineered strain KT2440 for biodegradation of xenobiotic pollutains, like bioremediation of naphthalene [46], carbofuran and chlorpyrifos [47], and 1,2-dichloroethane [39]. The salt tolerance of strain KT2440 is a desirable phenotype for bioconversion and bioremediation, especially for bioremediation exposed to highly saline wastewater or saline oil [48,49]. However, there are few reports about the salt tolerance of strain KT2440. The reannotation genome of *P. putida* KT2440 reveals the osmoregulatory metabolism and transport of osmolytes [50]. Strain KT2440 has several biosynthetic pathways of compatible molecules, including trehalose, glycine-betaine and proline. Additionally, some transporters involved in osmoregulation are located in the genome, such as I-type Na^+^/H^+^ antiporter NhaA-I, II-type Na^+^/H^+^ antiporter NhaA-II, Na^+^/H^+^ antiporter NhaB, K^+^ uptake system KdpCBAF, compatible solutes transporters ProP, major facilitator superfamily, BBCT family and CBC transporter. Unfortunately, there is little experimental evidence to verify the salt-tolerance function of the above genes. Borchert et al. identified several novel insights capable of increasing salt tolerance in strain KT2440, involving the GacA/GacS two-component system, the FleQ of flagellar synthesis, the Lap systems of cell surface adhesion and the DegP-like serine endoprotease [51]. Bojanovic et al. investigated the transcriptome of the strain KT2440 under high osmotic minimal salts medium [52]. They analyzed the differentially expressed RNAs and proposed main mechanisms against osmotic stress. However, there was no further experiment verification. There is still lack of comprehensive and systematic investigation into the mechanism of salt tolerance on strain KT2440.

In this study, we tested the salt tolerance performance of the environmental-type strain *P. putida* KT2440, and then analyzed the transcriptomic data between high-salinity culture and normal salinity culture to identify the endogenous salt-tolerant gene betaine-aldehyde dehydrogenase *betB*. The co-expression of Na^+^/H^+^ antiporter *EcnhaA* of *E. coli* obtained by screening exogenous salt-tolerant genes with *betB* further improved the salt-tolerant performance of the strain KT2440. The engineered strain was validated to degrade benzoic acid and protocatechuic acid under high-salinity conditions, while no biodegradation was detected in the normal KT2440 under the same conditions. This study presented the salt-tolerant elements and demonstrated the benefits of using synthetic biology strategies to construct a salt-resistant chassis to enhance bioremediation.

## 2. Materials and Methods

### 2.1. Strains and Culture Conditions

*E. coli* DH5α was used for plasmid construction and propagation. *E. coli* strains were inoculated into Luria–Bertani medium at 37 °C. *P. putida* strains were grown at 28 °C in LB medium, mineral salts medium (MSM) supplemented with 0.5% *w*/*v* glucose [53] or KB complex medium [54], the cmedium component purchased from Sinopharm Group Chemical Reagent Co., Ltd., Shanghai, China. If necessary, 40 mg/L tetracycline (Sangon Biotech, Shanghai, China)was added to the medium for plasmid maintain and 0.5 mM IPTG (Sangon Biotech, Shanghai, China) for inducing the expression of gene.

For the salt tolerance test, *P. putida* strains were streaked on a plate twice, then all colonies were transferred from the plate into a 250 mL flask containing 60 mL MSM for culture. After overnight culture for approximately 14 h, *P. putida* cells were harvested by centrifugation, washed twice with MSM, then inoculated into a 250 mL flask containing 60 mL MSM with NaCl, with a final OD_600_ at 0.02.

### 2.2. Transcriptome Analysis

The wild-type strain KT2440 was inoculated into KB medium with different NaCl concentrations, with 5% *w*/*v* NaCl added to the experimental group and no NaCl added to the control group. Each group was conducted in triplicate, and the logarithmic growth period was determined based on previous growth experiments. During the logarithmic growth period (24 h), 1 mL of culture medium was collected, centrifuged, and the supernatant was removed using an RNAase-free tip. The bacterial cells were frozen in liquid nitrogen for 15 min and stored at −80 °C. Shanghai Paisano Biotechnology Co. (Shanghai, China). was responsible for RNA-seq sequencing and preliminary analysis.

RNA-seq data were analyzed using various software packages. The Pearson correlation coefficient was used to assess the correlation of gene expression levels between samples. Principal component analysis and two-way cluster analysis were performed using DESeq (version 1.38.3) and Pheatmap (version 1.0.12) software packages, respectively. Differential gene expression was identified based on a log2 |fold change| ≥ 1 and a *p*-value ≤ 0.05. Volcano maps were drawn using the ggplots2 (version 3.4.1) package. Gene functional annotation and enrichment analysis were performed using the GO (https://www.geneontology.org/) and KEGG (https://www.kegg.jp/kegg/) databases. GO enrichment analysis was performed using the enrichment analysis tool TopGO (version 2.50.0), and a KEGG enrichment analysis was performed using the enrichment analysis tool KOBASO (version 3.0).

### 2.3. Quantitative Real-Time PCR (qRT-PCR) for Verification of Differentially Expressed Genes

*P. putida* KT2440 was streaked twice on plates and inoculated into KB medium with varying concentrations of NaCl. The experimental group consisted of 5% *w*/*v* NaCl, while the control group had no NaCl. Each group was tested in triplicate. Samples were collected during the logarithmic growth phase (24 h). RNA was extracted from the bacteria using the MolPure Bacterial RNA Kit (Yeasen Biotechnology, Shanghai, China), and cDNA was synthesized by reverse transcription using (4×) Hifair III SuperMix Plus (Yeasen Biotechnology, Shanghai, China). Fluorescent quantitative PCR amplification was performed using the qTOWER3G touch Real-Time PCR Thermal Cycler system (Analytik Jena AG, Jena, Germany). A PCR cycle consisting of 40 cycles was conducted with an initial step at 95 °C for 30 s followed by denaturation at 95 °C for 3 s and annealing/extension at 60 °C for 20 s. After the procedure, the lysis curve was examined, and an S-shaped curve and a Ct value between 20 and 30 indicated accurate quantitative analysis. The fold of relative change in transcript levels was calculated using the 2^−ΔΔCT^ method [55].

### 2.4. Gene Overexpression

Take overexpression of *betB* gene in *P. putida* KT2440 as an example: the genomic DNA of *P. putida* KT2440 was used as a template to amplify the gene *betB*, the standard PCR reaction involved 1 min at 98 °C; followed by 35 cycles of 20 s at 98 °C, 30 s at 58 °C, and 45 s (30 s/kb) at 72 °C; and finally 10 min at 72 °C. The plasmid pME6032 was digested with *Eco*R I, *Xho* I, and assembled with *betB* resulting pME6032-*betB*. The recombinant pME6032-*betB* was transformed into *P. putida* KT2440 by electroporation. The same method was used for overexpression of other genes. (Primers are listed in Supplementary Table S1).

### 2.5. Scanning Electron Micrograph

Strains were inoculated in MSM medium containing 0%, 4% or 5% *w*/*v* NaCl for 24 h, and then were centrifuged at 4800 rpm for 5 min. The precipitate was washed three times with PBS, resuspended with 0.5% glutaraldehyde at 4 °C for fixing 30 min. Then, the suspension was centrifuged and resuspended with 2.5% glutaraldehyde at 4 °C for fixing 12 h, and washed gradually with 50%, 70%, 100% ethanol. The suspension was then centrifuged, a small amount of cells was dropped onto filter paper, dried in a desiccator for 2 h and scraped onto a SEM column for gold spraying. The morphology of bacterium was observed by a scanning electron microscope (SEM) S3400II (Hitachi, Tokyo, Japan).

### 2.6. Biodegradation and Detection of Degradants

The degradants benzoic acid, catechol, and protocatechuic acid were added to MSM medium with varying NaCl concentrations at a final concentration of 2 mmol/L, and incubated at 28 °C and 200 rpm with shaking for degradation tests. A volume of 1 mL of culture was extracted at 0, 6, 12, 24, and 48 h and subjected to sample pretreatment. High-performance liquid chromatography (HPLC, Agilent 1260, Santa Clara, CA, USA) was used for detection: C18 reversed-phase column (Agilent Technologies, 5 μm, 4.6 × 250 mm, Santa Clara, CA, USA), methanol for phase A, and ammonium acetate (0.02 mol/L) for phase C. The flow rate was 1 mL/min, and the injection volume was 10 µL, with UV detection at a wavelength of 254 nm. The method used was 0–20 min, 80% of phase A, and 20% of phase C. The degradation rate is calculated as

$$\mathrm{degradation}\;\mathrm{rate}=\frac{\mathrm{original}\;\mathrm{concentration}-\mathrm{final}\;\mathrm{concentration}}{\mathrm{original}\;\mathrm{concentration}}\times 100\%$$

## 3. Results

### 3.1. The Salt Tolerance Performance of P. putida KT2440

**Figure 1:**
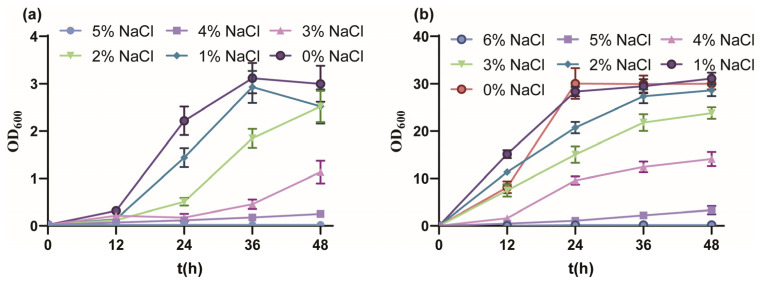
The growth curves of *P. putida* KT2440 in different conditions. (**a**) The growth curves of *P. putida* KT2440 in MSM medium supplemented with different concentrations of NaCl. (**b**) The growth curves of *P. putida* KT2440 in KB medium supplemented with different concentrations of NaCl. The data are the mean ± S.D. of three replicates.

Firstly, the salt tolerance performance of *P. putida* KT2440 cultured with MSM medium indicated a tolerated maximum of 4% *w*/*v* NaCl (Figure 1a). From 0% to 5% *w*/*v* NaCl, the final biomass gradually decreased and fell into a longer lag phase, and the OD_600_ value reached 3.00, 2.52, 2.52, 1.14, 0.250 and 0.022, respectively. The growth of strain KT2440 was drastically inhibited under 3% *w*/*v* NaCl, similar to the sublethal concentration of 0.5 M NaCl [51]. Due to the rich nutrition, the salt tolerance performance of strain KT2440 cultured with KB medium showed a tolerated maximum of 5% *w*/*v* NaCl (Figure 1b). From 0% to 6% *w*/*v* NaCl, the growth profile was similar to culture with MSM medium, and the OD_600_ value reached 30.03, 31.10, 28.26, 23.8, 14.10, 3.32 and 0.167, respectively. In summary, strain KT2440 tolerated moderate salinity.

### 3.2. Transcriptomic Analysis of P. putida KT2440 under NaCl Stress Conditions

In a previous study, it was found that strain KT2440 exhibited greater salt tolerance in KB medium than MSM and was able to withstand a stress pressure of 5% *w*/*v* NaCl concentration (Figure 1). We chose KB medium for salt tolerance screening of genes. Therefore, the experimental group of transcriptome is T (addition of 5% *w*/*v* NaCl) and the control group is C (no addition of NaCl).

#### 3.2.1. DEGs Analysis

Transcriptome analysis revealed 2568 genes that significantly changed in transcriptional level, with 1338 genes upregulated and 1230 genes downregulated after the addition of 5% *w*/*v* NaCl. The volcano plot of the transcriptome results showed the overall situation of differentially expressed genes upregulated and downregulated in the strain under hypertonic conditions (Supplementary Figure S1). Further information on the transcriptome could be found in Supplementary Table S2.

#### 3.2.2. Functional Annotation and Verification of DEGs

**Figure 2:**
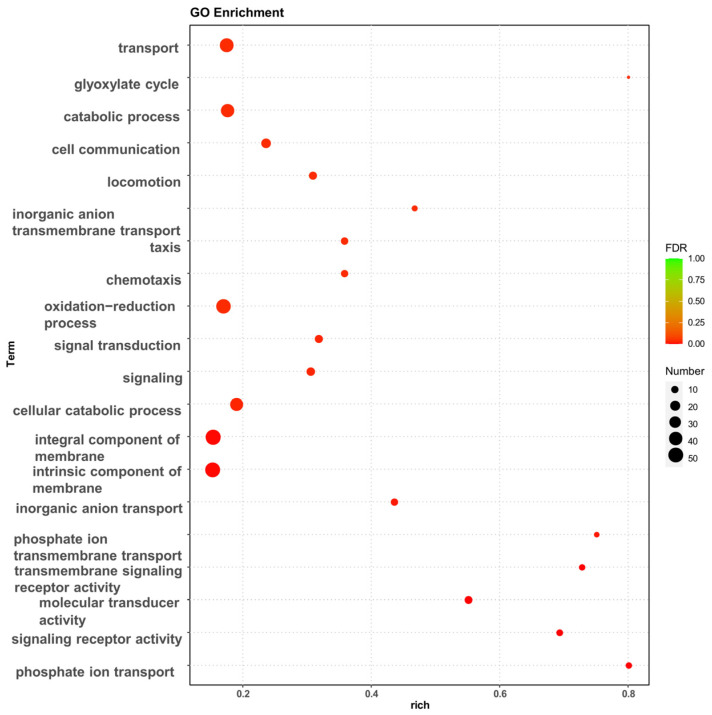
GO enrichment analysis of differentially expressed genes, bubble diagram of enrichment analysis. The horizontal coordinates are the rich factor, representing the number of differentially annotated genes in biological processes vs. the total number of annotated genes in biological processes. The vertical coordinates are biological processes, the size of the dots in the graph indicates the number of differential genes annotated in the corresponding biological process, and the shade of the color indicates the level of significance.

The effect of high osmotic stress on the strains may be visualized using Gene Ontology (GO) enrichment analysis and Kyoto Encyclopedia of Genes and Genomes (KEGG) enrichment analysis of DEGs. The GO enrichment analysis revealed that the experimental group of high osmotic pressure caused a significant increase in transcript levels in the strain, and the differentially expressed genes performed different biological functions and were widely distributed (Figure 2). In particular, a high number of differentially expressed genes were related to biological processes such as transport, membrane component, oxidation–reduction process, chemotaxis, and the cellular catabolic process.

The GO database categorizes gene functions into three categories: cellular component (CC), molecular function (MF), and biological process (BP). To evaluate the key biological tasks performed by differentially expressed genes, GO enrichment analysis was performed using topGO. Based on the three components of GO enrichment analysis, the number of enriched genes was 48 for cellular component, 404 for molecular function, and 901 for biological process. The top 10 GO terms were identified (Supplementary Figure S2).

The KEGG enrichment analysis of DEGs was performed to identify the metabolic pathways involved in salt-tolerance-related genes (Figure S3). It shows that high osmotic pressure significantly impacted the metabolic pathways of the strain. In particular, the significantly enriched metabolic pathways included bacterial chemotaxis, quorum sensing, ABC transporters, and a two-component system.

**Figure 3:**
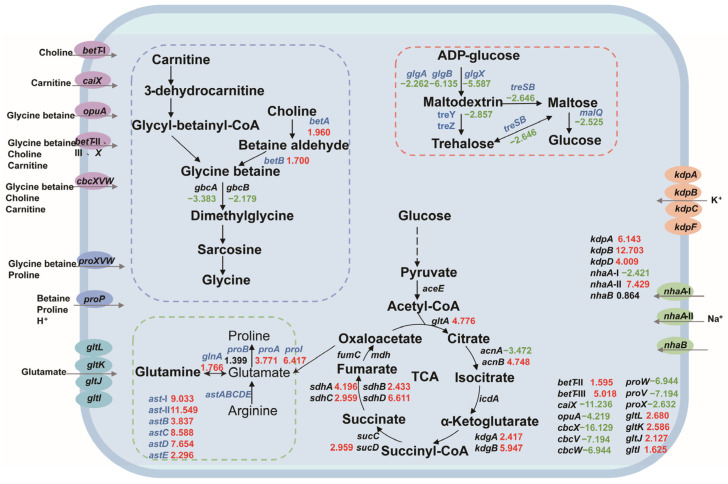
The changes in the central carbon metabolic network and the expression levels of salt-tolerant genes in *P. putida* KT2440. The numbers in the figure represent the multiple changes in the transcription level of the corresponding genes, with red indicating upregulation and green indicating downregulation. Blue genes represent the genes that this study focuses on. Dashed box: Correlation path of glycine-betaine synthesis (blue); amino acid-compatible solute synthesis-related pathways (green); pathways associated with trehalose synthesis (red). Ellipse: glycine-betaine, choline, carnitine channel protein (purple); proline, betaine channel protein (blue); glutamic acid channel protein (sky blue); potassium channel protein (orange); sodium channel protein (green).

Based on the salt tolerance mechanism, we constructed a central carbon metabolism network for *P. putida* KT2440 and analyzed the expression changes in salt-tolerance-related genes. Most of the genes involved in central carbon metabolism were upregulated, indicating that the strain needs to enhance this pathway for growth and survival under salt stress. Our analysis included the key components of betaine synthesis, amino acid metabolism, trehalose synthesis, compatible solute transport system, sodium–potassium ion transport system, and central carbon metabolism-related genes, as depicted in Figure 3.

Transcriptome analysis revealed potential salt tolerance gene targets in *P. putida* KT2440 under hypersaline conditions. Gene expression analysis identified several genes that were upregulated in the experimental group compared to the control group, indicating their potential involvement in salt tolerance. These genes included those involved in the transport system, ion pump, betaine, amino acid metabolism and molecular chaperone (Table 1).

**Figure 4:**
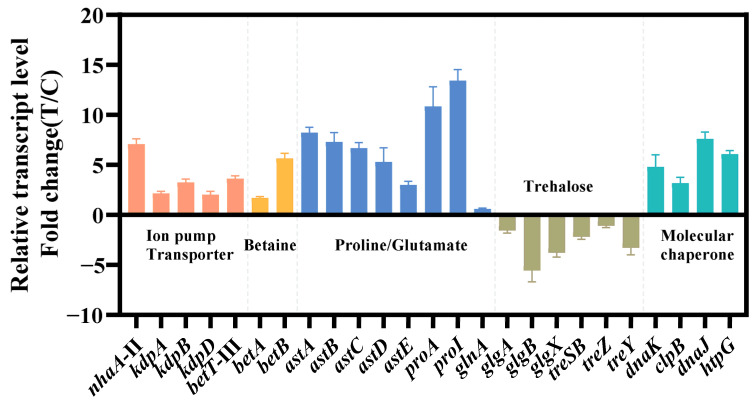
Transcriptional changes in genes involved in the transport system (red), ion pump (red), betaine synthesis (yellow), amino acid metabolism (blue), trehalose metabolism (brown) and molecular chaperone (green). The data at 24 h were provided. The data are the mean ± S.D. of three replicates.

The relevant genes were confirmed by qRT-PCR (Figure 4). The findings of qRT-PCR were largely consistent with the pattern observed in transcriptome data, which confirmed the reliability of transcriptome analysis. Next, we chose these differentially expressed genes to verify and improve the salt tolerance of strain KT2440.

### 3.3. Improve the Salt Tolerance of Strain KT2440

**Figure 5:**
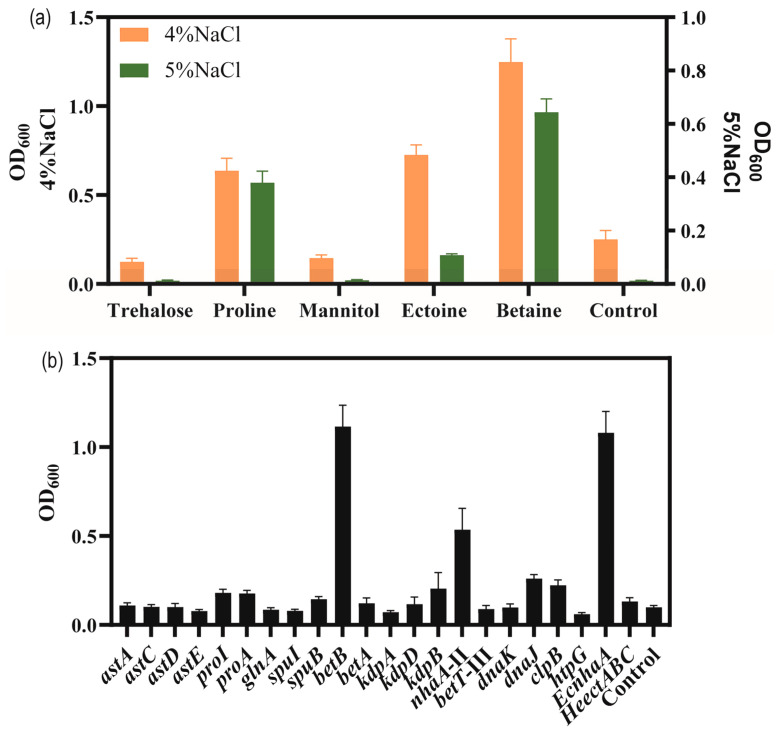
The growth status of strains in high-salinity MSM medium. (**a**) The effect of added compatible solute on the growth of *P. putida* KT2440 supplemented with high concentrations of NaCl. Cell density measured at 48 h. (**b**) The growth status of strains overexpressing salt-related genes under 4% *w*/*v* NaCl MSM medium. Cell density measured at 48 h.

To explore the adding effect of compatible solutes on the salt tolerance of strain KT2440, five different compatible solutes, trehalose, proline, mannitol, betaine and ectoine were, respectively, were supplemented with high-salinity MSM medium. As shown in Figure 5a, the biomass of the bacteria grown in MSM with added compatible solutes was higher than that of the control group supplemented with 4% *w*/*v* and 5% *w*/*v* NaCl concentrations. Among the five compatible solutes tested, betaine and proline showed the highest enhancement in salt tolerance performance. Compared to the control group, the addition of betaine and proline enhanced the biomass at 4% *w*/*v* NaCl by 154.4% and 188.1%, respectively. At 5% *w*/*v* NaCl, the biomass reached 0.64 and 0.38 for betaine and proline, respectively. Thus, with the addition of betaine or proline, the strain was able to tolerate 5% *w*/*v* NaCl.

In order to verify salt-tolerance-related genes, these upregulated-DEGs were overexpressed, including sodium–hydrogen antiporter protein gene *nhaA*-II of the sodium–potassium transport system, potassium ion transport system genes *kdpA*/*kdpB*, compatible solute betaine synthesis gene *betA*/*betB*, choline/carnitine/betaine transporter *betB*-III, glutamate synthesis gene cluster *astACDE*, proline synthesis gene *proA*/*proI*, and molecular chaperone-related genes *danK*/*dnaJ*/*clpB*/*htpG*. Additionally, some exogenous salt-tolerance-related genes such as the ectoine synthesis gene cluster *HeectABC* in *Halomonas elongata* DSM 2581^T^ [56] for achieving the complete biosynthesis of ectoine in strain KT2440, and the Na^+^/H^+^ antiporter protein gene *EcnhaA* in *E. coli* BL21 were also investigated [57].

Thus, a total of 18 strains overexpressing certain genes of interest in *P. putida* KT2440 were engineered to examine their potential for improving salt tolerance (Figure 5b). The results showed the biomass of overexpressing strains in MSM medium supplemented with 4% *w*/*v* NaCl for 48 h. To enhance the synthesis of glutamate, *astA*, *astC*, *astD*, and *astE* were overexpressed, respectively, and the growth of strain KT2440 did not increase under 4% *w*/*v* NaCl. The genes *proA* and *proI* were involved in proline biosynthesis and their overexpression increased the biomass to 0.177 and 0.181, under 4% *w*/*v* NaCl was compared to control, respectively. These results were similar to those obtained for the addition of proline. The genes *betAB* were involved in glycine-betaine biosynthesis. Overexpression of *betA* caused a limited effect of increasing the salt tolerance of strain KT2440. Overexpression of *betB* could be against high-salt stress and restore growth, resulting in a significant increase in the biomass to 1.12 compared to 0.099 for the control. These results were similar to those obtained for the addition of betaine. Overexpression of *kdpA* and *kdpB*, respectively, could not improve the salt tolerance of strain KT2440, due to the Kdp transporter consisting of four subunits. Overexpression of *nhaA*-II increased the biomass to 0.536. Overexpression of *betT*-III could not improve the salt tolerance of strain KT2440. Overexpression of endogenous molecular chaperone-related genes *danK*/*dnaJ*/*clpB*/*htpG* increased the biomass to 0.098, 0.260, 0.223 and 0.060, respectively. Fortunately, the heterologous expression of *EcnhaA* in strain KT2440 could boost growth to 1.08. Expression of *HeectABC* could improve biomass to 0.132. These results may indicate that glycine-betaine was a major osmolyte for strain KT2440.

**Figure 6:**
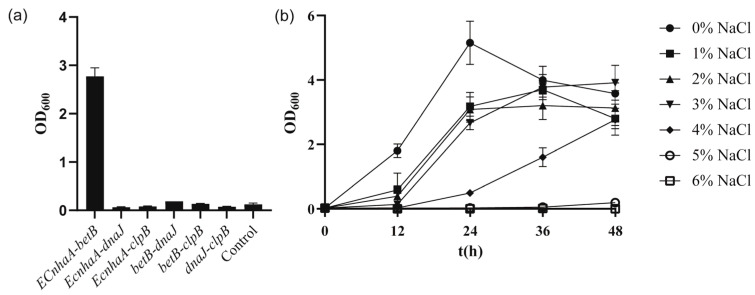
The growth status of strains with combinational overexpression in high-salinity MSM medium. (**a**) The biomass of strains with combinational overexpression at 48 h under 4% *w*/*v* NaCl MSM medium. (**b**) The growth curves of strain KT2440-*EcnhaA*-*betB* supplemented with different concentrations of NaCl. The data are the mean ± S.D. of three replicates.

To further improve the salt tolerance of the strain, *betB*, *dnaJ*, *clpB* and *EcnhaA* were selected for combinatorial expression. These six engineered strains (Figure 6a) exhibited different salt tolerance profiles under 4% *w*/*v* NaCl. Co-overexpression of *EcnhaA* and *betB* in strain KT2440 further enhanced the biomass to 2.77, compared with that for individual expression of *EcnhaA* and *betB*. The other five strains did not show a combination effect and hardly improved salt tolerance. We speculated that these combinations caused metabolic burden. The strain KT2440-*EcnhaA*-*betB* was evaluated under different concentrations of NaCl, and it was found that the maximum salinity tolerance of the strain increased to 5% *w*/*v* (Figure 6b) with a biomass of 0.193. These results indicated that together, the Na^+^/H^+^ transporter and betaine play a key role in the salt tolerance of strain KT2440. With the addition of proline and betaine (Figure S3), the strain KT2440-*EcnhaA*-*betB* could tolerate 6% *w*/*v* NaCl. This suggested that the salt tolerance of strain may be further improved by enhancing the synthesis of compatible solutes.

### 3.4. The Cell Morphology of the Salt-Tolerant Strain

**Figure 7:**
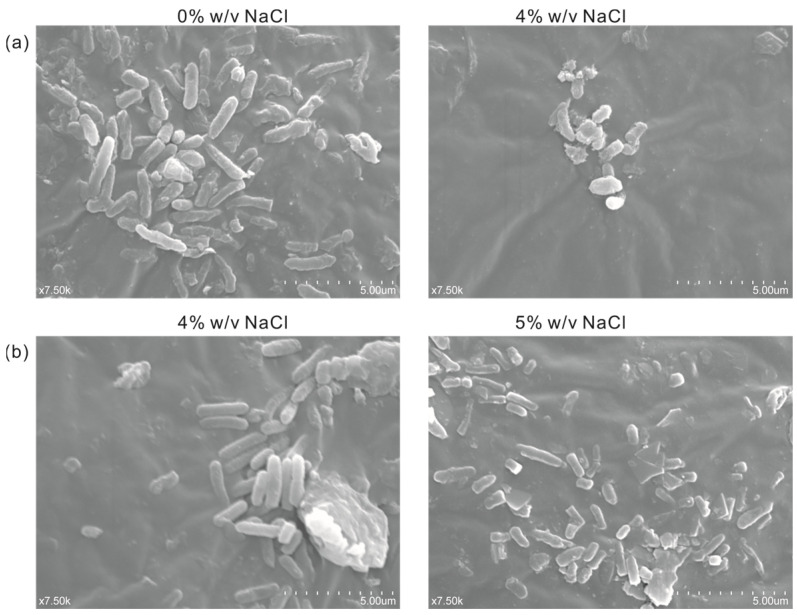
Scanning electron micrograph of strain KT2440 and KT2440-*EcnhaA*-*betB* under different concentrations of NaCl. (**a**) KT2440 cultured with 0% *w*/*v* NaCl or 4% *w*/*v* NaCl for 24 h, and then the cell morphology was determined. (**b**) KT2440-*EcnhaA*-*betB* cultured with 4% *w*/*v* NaCl or 5% *w*/*v* NaCl for 24 h, and then the cell morphology was determined.

In order to observe the difference in cell morphology between the engineered salt-tolerant strain and the normal strain KT2440 in response to salt stress, the salt-tolerant strain KT2440-*EcnhaA*-*betB* and the normal strain KT2440 were cultured with different NaCl concentrations. Thus, the cell morphology was observed by a scanning electron microscope according to the experimental method. As shown in Figure 7a, the normal strain KT2440 showed rod-shaped cells in the absence of salt stress (0% *w*/*v* NaCl). While exposed to high-salt stress (4% *w*/*v* NaCl), its cells showed short rods. Due to the impossibility of the normal strain to tolerate 4% *w*/*v* NaCl, the loss of intracellular water in the hypertonic state leads to cell atrophy. The cell morphology of strain KT2440-*EcnhaA*-*betB* was not affected by 4% *w*/*v* NaCl (Figure 7b), and still maintained the normal morphology of rod shape. Some of the cells also had a short rod shape under 5% *w*/*v* NaCl. These results showed that the engineered strain KT2440-*EcnhaA*-*betB* had a better growth condition and improved its salt tolerance compared with the normal strain KT2440 at 4% *w*/*v* NaCl.

### 3.5. The Degradation Properties of Engineered Strains

**Figure 8:**
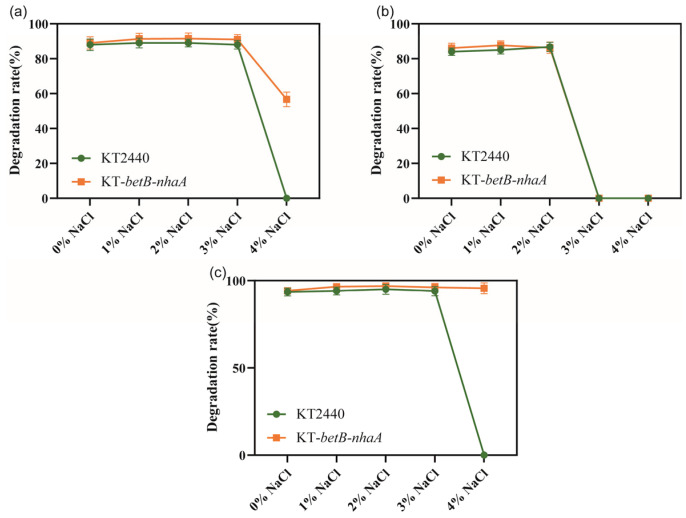
Comparison of the degradation rates of aromatic compounds between salt-tolerant strain KT2440-*EcnhaA-betB* and strain KT2440 supplemented with different concentrations of NaCl. (**a**) Degradation of benzoic acid. (**b**) Degradation of catechol. (**c**) Degradation of protocatechuic acid. The degradation rates were measured at 48 h.

We investigated whether the salt-tolerant strain KT2440-*EcnhaA-betB* exhibited changes in its ability to degrade aromatic compounds. Firstly, we evaluated the degradation rate of three aromatic compounds (benzoic acid, catechol, and protocatechuicacid) using the normal strain KT2440 supplemented with different NaCl concentrations. Then, we separately analyzed the degradation ability of strain KT2440-*EcnhaA-betB* towards the same three aromatic compounds under identical conditions. We compared the maximum 48 h degradation rates of both strains (Figure 8). According to the degradation results, there was no significant difference in the degradation rates of strain KT2440-*EcnhaA-betB* and strain KT2440 for the three tested aromatic compounds under 0–3% *w*/*v* NaCl. However, under 4% *w*/*v* NaCl, strain KT2440-*EcnhaA-betB* exhibited a maximum degradation rate of 56.70% and 95.64% for benzoic acid and protocatechuic acid, respectively, after 48 h of incubation in a medium with only the aromatic compound as the carbon source. These results indicate that the engineered salt-tolerant strain KT2440-*EcnhaA*-*betB* was able to degrade aromatic compounds in high-salt environments.

## 4. Discussion

*P. putida* KT2440 exhibited different salt tolerance profiles in MSM medium and KB medium. The strain KT2440 could maximally tolerate up to 4% *w*/*v* NaCl MSM medium (Figure 1). Due to the nutrient and carbon source-rich KB medium being more conducive to synthesizing compatible solutes, the strain KT2440 further tolerated up to 5% *w*/*v* NaCl. The transcriptome of the strain KT2440 under 5% *w*/*v* NaCl KB medium compared with KB medium without NaCl was conducted, and DEGs were analyzed. The GO and KEGG (Figure 2, Figures S1 and S2) enrichment analysis of DEGs revealed that high osmotic pressure significantly affected the cell membrane components and ion transport across the membrane of the strain. This resulted in a stress response in the strain, which further impacted intracellular signal transduction and cellular communication movements. Additionally, the hypertonic conditions also affected the intracellular catabolic levels of the strain.

Microbes effectively maintain osmotic pressure balance by incorporating compatible solutes like trehalose, glycine, betaine ectoine, proline, and other polar soluble molecules [33]. In terms of the addition of various compatible solutes, including trehalose, proline, manniol, ectoine and betaine, wherein proline, ectoine and bataine enhanced the growth of KT2440 under 4% or 5% *w*/*v* NaCl MSM medium, the GO and KEGG enrichment analysis of DEGs in *P. putida* KT2440 under hypertonic conditions revealed the upregulated expression of the biosynthesis genes of proline and betaine, such as *astABCDE*, *proAI* and *betAB*. This was followed by overexpression of critical genes associated with the biosynthesis of proline, ectoine and betaine, showing that only the overexpression of betaine-aldehyde dehydrogenase *betB* improved the growth of the strain KT2400 under 4% *w*/*v* NaCl MSM medium. A previous study indicates that the strain KT2440 rapidly accumulates ectoine as a “temporary” emergency response, with cells adapting to osmotic stress by synthesizing betaine instead of ectoine [58]. We speculated that betaine is the major compatible solute of the strain KT2440.

On the other hand, microbes utilize membrane transporters to transport salt ions to regulate osmolarity. The Kdp and Trk transporter systems are involved in osmoregulation of K^+^ uptake in many organisms [59]. The transcriptome data showed that the expression levels of the *kdpA*, *kdpB*, and *kdpD* genes involved in the Kdp transport system were upregulated under hypertonic conditions (Table 1, Figure 4). The expression levels of the *trkA* and *trkH* genes involved in the Trk transport system were not significantly changed under hypertonic conditions (Table S2). The individual overexpression of *kdpA*, *kdpB* and *kdpD* in KT2440 failed to improve the growth cultured with 4% *w*/*v* NaCl MSM medium. We speculate that intracellular accumulation of K^+^ may not be a salt tolerance strategy for KT2440. The Na^+^/H^+^ reverse transporter protein is a type of transporter protein present in the cell membrane of both eukaryotes and prokaryotes [22]. The *nhaA*-I, *nhaA*-II and *nhaB* reverse transporter proteins are located in the strain KT2440, where *nhaA*-II was significantly upregulated by a 7.429-fold under hypertonic conditions (Table 1). The overexpression of *nhaA*-II in KT2440 could slightly improve the growth cultured with 4% *w*/*v* NaCl MSM medium (Figure 5b). This indicated that *nhaA*-II may against salt tolerance in the strain KT2440. The overexpression of *EcnhaA* in KT2440 could significantly improve the growth cultured with 4% *w*/*v* NaCl MSM medium (Figure 5b). This suggested that *EcnhaA* was an efficient Na^+^/H^+^ reverse transporter protein in strain KT2440.

Molecular chaperones assist protein folding to improve strain survival in response to various stressors. Based on the transcriptome information, *dnaK*, *dnaJ*, *clpB* and *htpG* were selected for overexpression validation, where *dnaJ* and *clpB* slightly improved the growth of the strain under 4% *w*/*v* NaCl MSM medium (Figure 5b). The *betB*, *EcnhaA*, *dnaJ* and *clpB* were selected for combinatorial expression. Co-overexpression of *EcnhaA* and *betB* in the strain KT2440 further enhanced the strain growth under 4% *w*/*v* NaCl MSM medium and the maximum salinity tolerance of the strain KT2440 increased to 5% *w*/*v* (Figure 6b). Scanning electron microscopy showed that the engineered strain KT2440-*EcnhaA*-*betB* showed normal morphology at 4% NaCl and some cells had a short rod shape at 5% NaCl. In short, overexpression of *EcnhaA* and *betB* conferred salt tolerance resistance to the strain KT2440.

The salt-tolerant strain KT2440-*EcnhaA*-*betB* exhibited degradation of aromatic compounds in high-salinity environments. Benzoic acid and protocatechuic acid were degraded under 4% *w*/*v* NaCl, while no biodegradation was detected in the normal KT2440 under the same conditions. Under 4% *w*/*v* NaCl, the strain KT2440-*EcnhaA*-*betB* exhibited a degradation rate of 56.70% and 95.64% for benzoic acid and protocatechuic acid, respectively. However, the biodegradation of catechol by strain KT2440 or KT2440-*EcnhaA-betB* failed when the salt concentration exceeded 2% *w*/*v* NaCl. This suggests that high salinity reduced the solubility of catechol to satisfy the growth. Further validation of the biodegradation process could be conducted by isotope labelling experiments in the future. In summary, these findings suggest that strain KT2440-*EcnhaA*-*betB* may have potential for the biodegradation of aromatic compounds in high-salinity environments.

## 5. Conclusions

Herein, two salt-resistant elements, betaine-aldehyde dehydrogenase *betB* from *P. putida* KT2440 and the Na^+^/H^+^ antiporter *EcnhaA* from *E. coli*, could individually endow KT2440 to tolerate 4% *w*/*v* NaCl MSM medium. Co-expression of *betB* and *EcnhaA* enabled a salt-tolerant strain KT2440-*EcnhaA-betB* to tolerate 5% *w*/*v* NaCl MSM medium. More importantly, the modified strain KT2440-*EcnhaA*-*betB* was able to degrade selected pollutants under 4% *w*/*v* NaCl. Thus, a potential salt-tolerant bioremediation platform strain KT2440-*EcnhaA*-*betB* was constructed. This study provides a case study for the engineering of salt tolerance in environmental microorganisms. These two elements may be able to improve the salt tolerance of typical environmental microorganisms (e.g., *Alcaligenes* and *Rhodococcus*) and to construct microbial communities for remediation of multiple pollutants.

In the future, we hope that the salt-tolerant bioremediation platform strain KT2440-*EcnhaA-betB* could be applied to degrade pollutants in hypersaline environments and more salt-tolerant-related genes could be studied to improve the tolerance of environmental microorganisms.

## Figures and Tables

**Table 1:** Transcriptome data of highly related genes.

Gene ID	Gene Name	Fold Change (T/C)	*p*-Value	Description
PP_4158	*kdpD*	4.009	0.0003	Sensor protein KdpD
PP_4160	*kdpB*	12.703	0.0000	K^+^ transporting ATPase, KdpB subunit
PP_4161	*kdpA*	6.143	0.0087	Potassium-transporting ATPase A chain
PP_3958	*nhaA*-II	7.429	0.0000	Na^+^/H^+^ antiporter NhaA-II
PP_3957	*betB*-III	5.018	0.0000	Choline/carnitine/betaine transporter
PP_5064	*betA*	1.960	0.0348	Choline dehydrogenase
PP_5063	*betB*	1.700	0.0878	NAD-dependent betaine aldehyde dehydrogenase
PP_4479	*astA*	9.033	0.0000	Arginine N-succinyltransferase
PP_4477	*astB*	3.837	0.0000	N-succinylarginine dihydrolase
PP_4481	*astC*	8.588	0.0000	Succinylornithine transaminase/acetylornithine aminotransferase
PP_4478	*astD*	7.654	0.0000	N-succinylglutamate 5-semialdehyde
PP_4475	*astE*	2.296	0.0044	Succinylglutamate desuccinylase
PP_5095	*proI*	6.417	0.0012	Pyrroline-5-carboxylate reductase
PP_4811	*proA*	3.770	0.0000	Gamma-glutamyl phosphate reductase
PP_5184	*spuI*	2.212	0.0072	Glutamylpolyamine synthetase
PP_5046	*glnA*	1.776	0.0386	Glutamine synthetase
PP_4727	*dnaK*	3.217	0.0000	Molecular chaperone DnaK
PP_0625	*clpB*	2.507	0.0564	ATP-dependent chaperone ClpB
PP_4726	*dnaJ*	4.255	0.0000	Molecular chaperone DnaJ
PP_4179	*htpG*	5.356	0.0000	Molecular chaperone HtpG

## Data Availability

The data presented in this study are available from the authors.

## Supplementary Materials

The following supporting information can be downloaded at: https://www.mdpi.com/article/10.3390/biology13060404/s1.

## References

[1] Vreeland R.H., Hochstein L.I. (1992). The Biology of Halophilic Bacteria.

[2] Oren A. (2008). Microbial life at high salt concentrations: Phylogenetic and metabolic diversity. Saline Syst..

[3] Padan E., Venturi M., Gerchman Y., Dover N. (2001). Na^+^/H^+^ antiporters. Biochim. Biophs. Acta..

[4] Wani A.K., Akhtar N., Sher F., Navarrete A.A., Américo-Pinheiro J.H.P. (2022). Microbial adaptation to different environmental conditions: Molecular perspective of evolved genetic and cellular systems. Arch. Microbiol..

[5] Kumar S., Paul D., Bhushan B., Wakchaure G.C., Meena K.K., Shouche Y. (2020). Traversing the “Omic” landscape of microbial halotolerance for key molecular processes and new insights. Crit. Rev. Microbiol..

[6] Gunde-Cimerman N., Plemenitas A., Oren A. (2018). Strategies of adaptation of microorganisms of the three domains of life to high salt concentrations. Fems. Microbiol. Rev..

[7] Ahmad E., Sharma S.K., Kashyap A.S., Manzar N., Sahu P.K., Singh U.B., Singh H.V., Sharma P.K. (2023). Evaluation of osmotolerant potential of *Halomonas sulfidaeris* MV-19 isolated from a mud volcano. Curr. Microbiol..

[8] Goswami S.K., Kashyap A.S., Kumar R., Gujjar R.S., Singh A., Manzar N. (2023). Harnessing rhizospheric microbes for eco-friendly and sustainable crop production in saline environments. Curr. Microbiol..

[9] Dimroth P., Jockel P., Schmid M. (2001). Coupling mechanism of the oxaloacetate decarboxylase Na^+^ pump. Biochim. Biophs. Acta..

[10] Lietzan A.D., St Maurice M. (2014). Functionally diverse biotin-dependent enzymes with oxaloacetate decarboxylase activity. Arch. Biochem. Biophys..

[11] Vitt S., Prinz S., Hellwig N., Morgner N., Ermler U., Buckel W. (2020). Molecular and Low-Resolution Structural Characterization of the Na^+^-Translocating Glutaconyl-CoA Decarboxylase From. Front. Microbiol..

[12] Becher B., Muller V., Gottschalk G. (1992). The Methyl-Tetrahydromethanopterin-Coenzyme-M Methyltransferase of Methanosarcina Strain Go1 Is a Primary Sodium-Pump. Fems. Microbiol. Lett..

[13] Takase K., Kakinuma S., Yamato I., Konishi K., Igarashi K., Kakinuma Y. (1994). Sequencing and characterization of the ntp gene cluster for vacuolar-type Na(+)-translocating ATPase of *Enterococcus hirae*. J. Biol. Chem..

[14] Verkhovsky M.I., Bogachev A.V. (2010). Sodium-translocating NADH:quinone oxidoreductase as a redox-driven ion pump. Biochim. Biophs. Acta.

[15] Yang L.F., Jiang J.Q., Zhang B., Zhao B.S., Wang L., Yang S.S. (2006). A primary sodium pump gene of the moderate halophile exhibits secondary antiporter properties. Biochem. Biophys. Res. Commun..

[16] Mager T., Braner M., Kubsch B., Hatahet L., Alkoby D., Rimon A., Padan E., Fendler K. (2013). Differential effects of mutations on the transport properties of the Na^+^/H^+^ antiporter NhaA from *Escherichia coli*. J. Biol. Chem..

[17] Padan E. (2014). Functional and structural dynamics of NhaA, a prototype for Na^+^ and H^+^ antiporters, which are responsible for Na^+^ and H^+^ homeostasis in cells. Biochim. Biophs. Acta..

[18] Pinner E., Kotler Y., Padan E., Schuldiner S. (1993). Physiological role of nhaB, a specific Na^+^/H^+^ antiporter in *Escherichia coli*. J. Biol. Chem..

[19] Prágai Z., Eschevins C., Bron S., Harwood C.R. (2001). Bacillus subtilis NhaC, an Na^+^/H^+^ antiporter, influences expression of the *phoPR* operon and production of alkaline phosphatases. J. Bacteriol..

[20] Dzioba J., Ostroumov E., Winogrodzki A., Dibrov P. (2002). Cloning, functional expression in *Escherichia coli* and primary characterization of a new Na^+^/H^+^ antiporter, NhaD, of *Vibrio cholerae*. Mol. Cell. Biochem..

[21] Patiño-Ruiz M., Ganea C., Calinescu O. (2022). Prokaryotic Na^+^/H^+^ exchangers—Transport mechanism and essential residues. Int. J. Mol. Sci..

[22] Xu N., Cheng H., Liu Q., Liu J., Ma Y. (2015). Research progress of the Na^+^/H^+^ antiporters in bacteria. Microbiol. China.

[23] Wang W., Tang H., Xu P. (2015). Salt-tolerance related genes in halophilic bacteria and archaea. Microbiology China.

[24] Edbeib M.F., Wahab R.A., Huyop F. (2016). Halophiles: Biology, adaptation, and their role in decontamination of hypersaline environments. World J. Microb. Biotechnol..

[25] Roberts M.F. (2005). Organic compatible solutes of halotolerant and halophilic microorganisms. Saline Syst..

[26] Waditee R., Tanaka Y., Aoki K., Hibino T., Jikuya H., Takano J., Takabe T., Takabe T. (2003). Isolation and Functional Characterization of N-Methyltransferases That Catalyze Betaine Synthesis from Glycine in a Halotolerant Photosynthetic Organism *Aphanothece halophytica*. J. Biol. Chem..

[27] Brill J., Hoffmann T., Bleisteiner M., Bremer E. (2011). Osmotically Controlled Synthesis of the Compatible Solute Proline Is Critical for Cellular Defense of against High Osmolarity. J. Bacteriol..

[28] Wood J.M., Bremer E., Csonka L.N., Kraemer R., Poolman B., van der Heide T., Smith L.T. (2001). Osmosensing and osmoregulatory compatible solute accumulation by bacteria. Comp. Biochem. Physiol. A Mol. Integr. Physiol..

[29] Zahid N., Schweiger P., Galinski E., Deppenmeier U. (2015). Identification of mannitol as compatible solute in *Gluconobacter oxydans*. Appl. Microbiol. Biot..

[30] Galleguillos P.A., Grail B.M., Hallberg K.B., Demergasso C.S., Johnson D.B. (2018). Identification of trehalose as a compatible solute in different species of acidophilic bacteria. J. Microbiol..

[31] Kadam P., Khisti M., Ravishankar V., Barvkar V., Dhotre D., Sharma A., Shouche Y., Zinjarde S. (2024). Recent advances in production and applications of ectoine, a compatible solute of industrial relevance. Bioresour. Technol..

[32] Fenizia S., Thume K., Wirgenings M., Pohnert G. (2020). Ectoine from Bacterial and Algal Origin Is a Compatible Solute in Microalgae. Mar. Drugs.

[33] Ventosa A., Nieto J.J., Oren A. (1998). Biology of moderately halophilic aerobic bacteria. Microbiol. Mol. Biol. Rev..

[34] Papoušková K., Sychrová H. (2007). Production of *Yarrowia lipolytica* Nha2 Na^+^/H^+^ antiporter improves the salt tolerance of *Saccharomyces cerevisiae*. Folia Microbiol..

[35] Sanadhya P., Agarwal P., Khedia J., Agarwal P.K. (2015). A low-affinity K^+^ transporter *AlHKT2;1* from recretohalophyte aeluropus lagopoides confers salt tolerance in yeast. Mol. Biotechnol..

[36] Willscher S., Jablonski L., Fona Z., Rahmi R., Wittig J. (2017). Phytoremediation experiments with *Helianthus tuberosus* under different pH and heavy metal soil concentrations. Hydrometallurgy.

[37] Tang L., Hamid Y., Zehra A., Sahito Z.A., He Z., Hussain B., Gurajala H.K., Yang X. (2019). Characterization of fava bean (*Vicia faba* L.) genotypes for phytoremediation of cadmium and lead co-contaminated soils coupled with agro-production. Ecotoxicol. Environ. Saf..

[38] Mishra S., Lin Z., Pang S., Zhang W., Bhatt P., Chen S. (2021). Recent advanced technologies for the characterization of xenobiotic-degrading microorganisms and microbial communities. Front. Bioeng. Biotechnol..

[39] Huo K.Y., Wang S.Q., Zhao W.W., Guo H.F., Xiong W.N., Liu R.H., Yang C. (2023). Creating an efficient 1,2-dichloroethane-mineralizing bacterium by a combination of pathway engineering and promoter engineering. Sci. Total. Environ..

[40] Martínez-García E., de Lorenzo V. (2017). Molecular tools and emerging strategies for deep genetic/genomic refactoring of *Pseudomonas*. Curr. Opin. Biotechnol..

[41] Martinez-Garcia E., de Lorenzo V. (2023). *Pseudomonas putida* as a synthetic biology chassis and a metabolic engineering platform. Curr. Opin. Biotechnol..

[42] Nikel P.I., de Lorenzo V. (2018). *Pseudomonas putida* as a functional *chassis* for industrial biocatalysis: From native biochemistry to *trans*-metabolism. Metab. Eng..

[43] Ma C., Mu Q.X., Xue Y.B., Xue Y.F., Yu B., Ma Y.H. (2021). One major facilitator superfamily transporter is responsible for propionic acid tolerance in *Pseudomonas putida* KT2440. Microb. Biotechnol..

[44] Zou L.H., Jin X.Z., Tao Y.M., Zheng Z.J., Ouyang J. (2022). Unraveling the mechanism of furfural tolerance in engineered *Pseudomonas putida* by genomics. Front. Microbiol..

[45] Mohamed E.T., Werner A.Z., Salvachúa D., Singer C.A., Szostkiewicz K., Jiménez-Díaz M.R., Eng T., Radi M.S., Simmons B.A., Mukhopadhyay A. (2020). Adaptive laboratory evolution of *Pseudomonas putida* KT2440 improves *p*-coumaric and ferulic acid catabolism and tolerance. Metab. Eng. Commun..

[46] Fernández M., Niqui-Arroyo J.L., Conde S., Ramos J.L., Duque E. (2012). Enhanced tolerance to naphthalene and enhanced rhizoremediation performance for Pseudomonas putida KT2440 via the NAH7 catabolic plasmid. Appl. Environ. Microb..

[47] Gong T., Liu R.H., Che Y., Xu X.Q., Zhao F.J., Yu H.L., Song C.J., Liu Y.P., Yang C. (2016). Engineering *Pseudomonas putida* KT2440 for simultaneous degradation of carbofuran and chlorpyrifos. Microb. Biotechnol..

[48] Zhao Y.Y., Zhuang X.M., Ahmad S., Sung S., Ni S.Q. (2020). Biotreatment of high-salinity wastewater: Current methods and future directions. World J. Microbiol. Biotechnol..

[49] Feng H.J., Chen L., Ding Y.C., Ma X.J., How S.W., Wu D. (2022). Mechanism on the microbial salt tolerance enhancement by electrical stimulation. Bioelectrochemistry.

[50] Belda E., van Heck R.G.A., Lopez-Sanchez M.J., Cruveiller S., Barbe V., Fraser C., Klenk H.P., Petersen J., Morgat A., Nikel P.I. (2016). The revisited genome of *Pseudomonas putida* KT2440 enlightens its value as a robust metabolic *chassis*. Environ. Microbiol..

[51] Borchert A.J., Bleem A., Beckham G.T. (2023). RB-TnSeq identifies genetic targets for improved tolerance of *Pseudomonas putida* towards compounds relevant to lignin conversion. Metab. Eng..

[52] Bojanovic K., D’Arrigo I., Long K.S. (2017). Global Transcriptional Responses to Osmotic, Oxidative, and lnnipenem Stress Conditions in. Appl. Environ. Microbiol..

[53] Li Q.G., Wang X.Y., Yin G.B., Gai Z.H., Tang H.Z., Ma C.Q., Deng Z.X., Xu P. (2009). New metabolites in dibenzofuran cometabolicdegradation by a biphenyl-cultivated *Pseudomonas putida* srain B6-2. Environ. Sci. Technol..

[54] Wang S.W., Bilal M., Zong Y.N., Hu H.B., Wang W., Zhang X.H. (2018). Development of a Plasmid-Free Biosynthetic Pathway for Enhanced Muconic Acid Production in *Pseudomonas chlororaphis* HT66. Acs Synth. Biol..

[55] Livak K.J., Schmittgen T.D. (2001). Analysis of relative gene expression data using real-time quantitative PCR and the 2^−ΔΔCT^ method. Methods.

[56] Schwibbert K., Marin-Sanguino A., Bagyan I., Heidrich G., Lentzen G., Seitz H., Rampp M., Schuster S.C., Klenk H.P., Pfeiffer F. (2011). A blueprint of ectoine metabolism from the genome of the industrial producer Halomonas elongata DSM 2581^T^. Environ. Microbiol..

[57] Padan E., Tzubery T., Herz K., Kozachkov L., Rimon A., Galili L. (2004). NhaA of *Escherichia coli*, as a model of a pH-regulated Na^+^/H^+^antiporter. Biochim. Biophs. Acta.

[58] He J., Jiang J., Jia K., Huang X., Li S. (2006). Glycine betaine supplied exogenously enhance salinity tolerance of *Pseudomonas putida* DLL-1. Wei Sheng Wu Xue Bao.

[59] Gralla J.D., Vargas D.R. (2006). Potassium glutamate as a transcriptional inhibitor during bacterial osmoregulation. EMBO J..

