# Supplementary material for: Improvement in Salt Tolerance Ability of Pseudomonas putida KT2440
Source: Biology (Basel). 2024 Jun 1;13(6):404. doi: 10.3390/biology13060404 (PMC11200750; doi:10.3390/biology13060404)
Supplement: Supplementary file 1 [file biology-13-00404-s001.zip › Supplemental Material/Supplementary materials FigureS1-S4 & Table S1&S3.pdf]

Supporting Information for

Analysis and Improvement of Salt Tolerance Ability in *Pseudomonas putida* KT2440

Min Fan[1#], Shuyu Tan[1#], Wei Wang[1], Xuehong Zhang[1]*

1 State Key Laboratory of Microbial Metabolism, School of Life Sciences and Biotechnology, Shanghai Jiao Tong University

[#] Min Fan and Shuyu Tan contributed equally to this work.

[*]Corresponding author: Tel.: +86-21-3420-7047; Fax: +86-21-3420-5081

E-mail: xuehzhang@sjtu.edu.cn

Figure S1 RNA-seq volcano diagram.

Figure S2 GO enrichment analysis of differentially expressed genes.

Figure S3 KEGG enrichment analysis of differentially expressed genes.

Figure S4 Comparison of growth conditions between strain KT2440-Ec*nhaA-betB* and wild-type strain KT2440.

Table S1 Primers used in this study.

Table S3 Sequence of *Heect* cluster.

Figure S1 RNA-seq volcano diagram. In the figure, the vertical dotted line is the 2x expression difference threshold, and the horizontal dotted line is the P-value=0.05 threshold. Each dot represents a gene, with red representing up-regulated genes, blue down-regulated genes, and gray representing non-significantly differentially expressed genes.

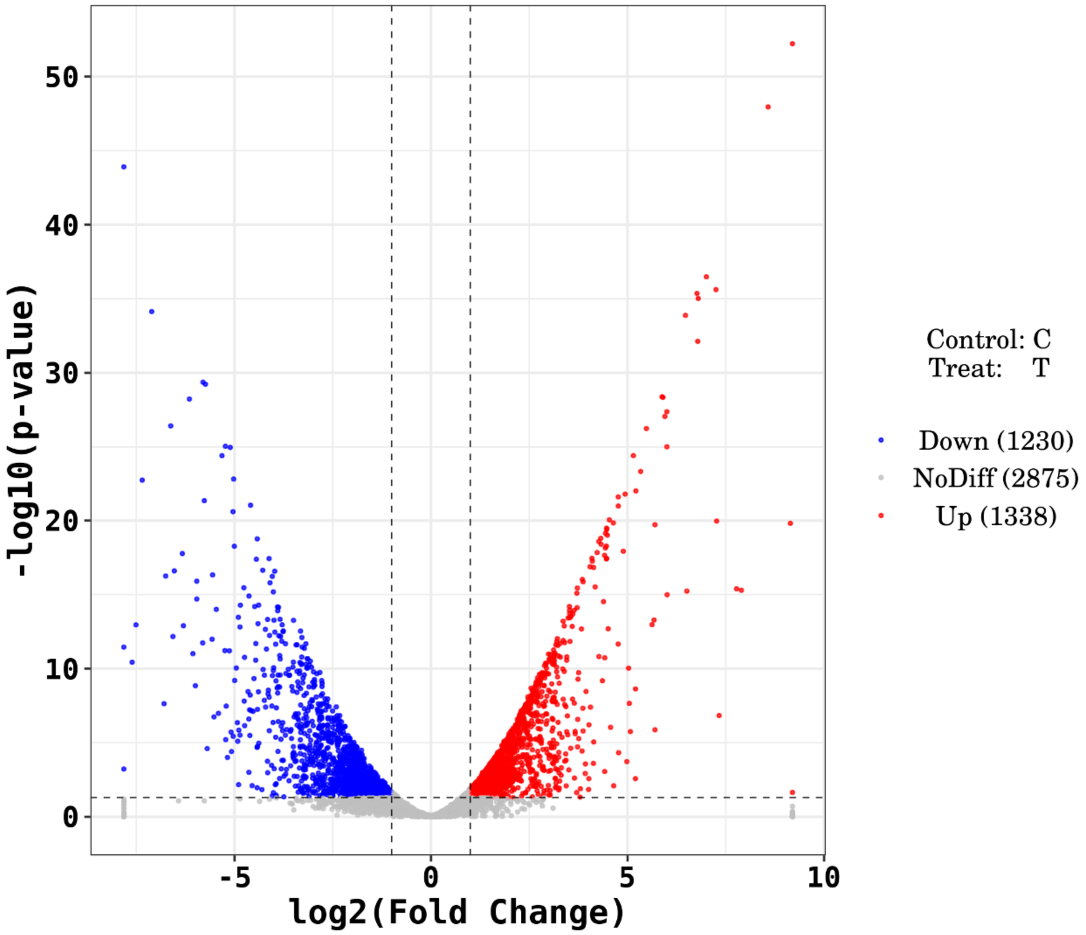

Figure S2 GO enrichment analysis of differentially expressed genes. Enrichment analysis of three subcategories. CC, Cellular component; MF, Molecular function; BP, Biological process.

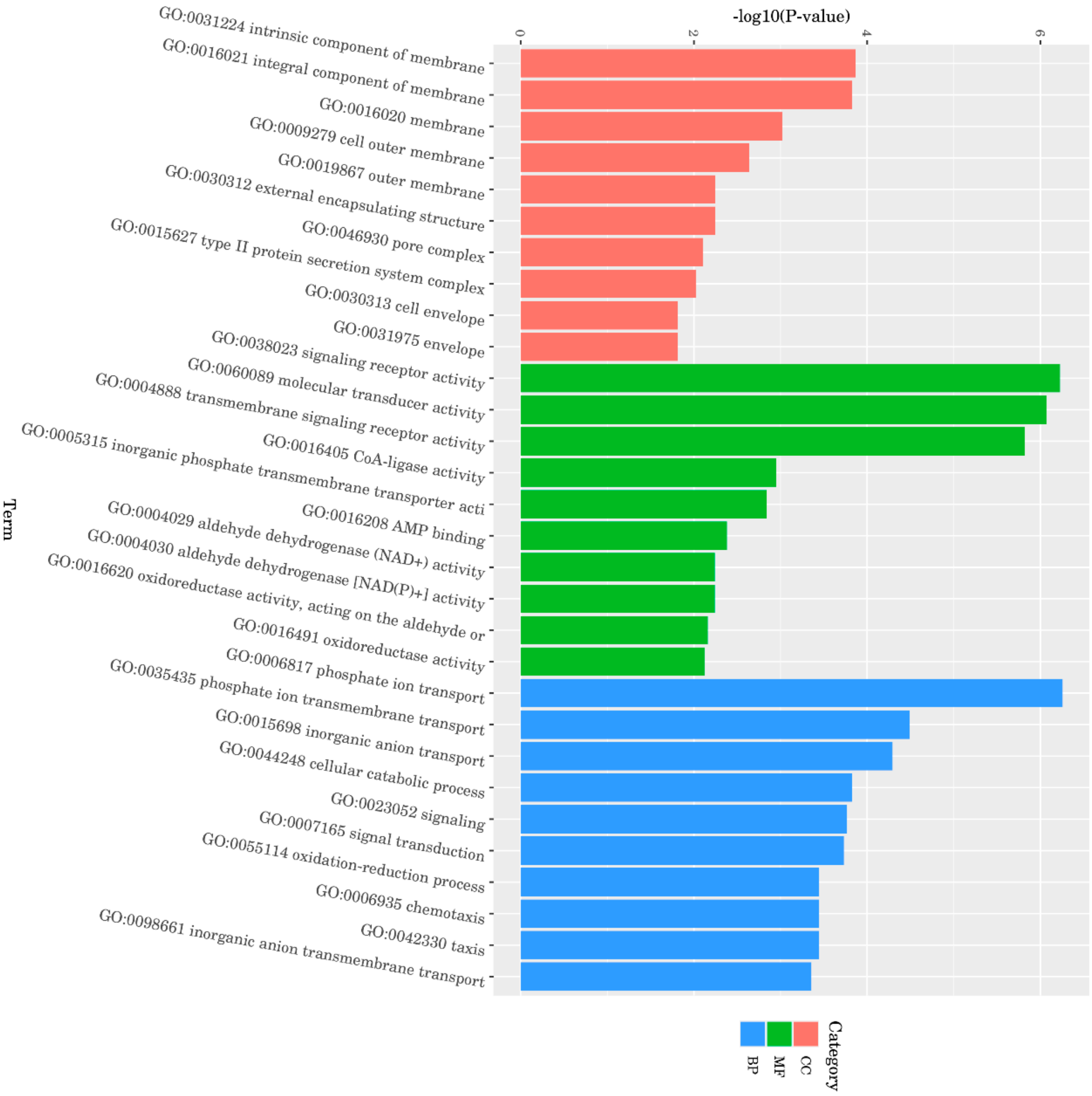

Figure S3 KEGG enrichment analysis of differentially expressed genes, bubble diagram of enrichment analysis. The horizontal coordinates are rich factor, represented number of differentially annotated genes in pathway vs total number of annotated genes in pathway. The vertical coordinates are pathway, the size of the dots in the graph indicates the number of differential genes annotated in the corresponding pathway, and the shade of the colour indicates the level of significance.

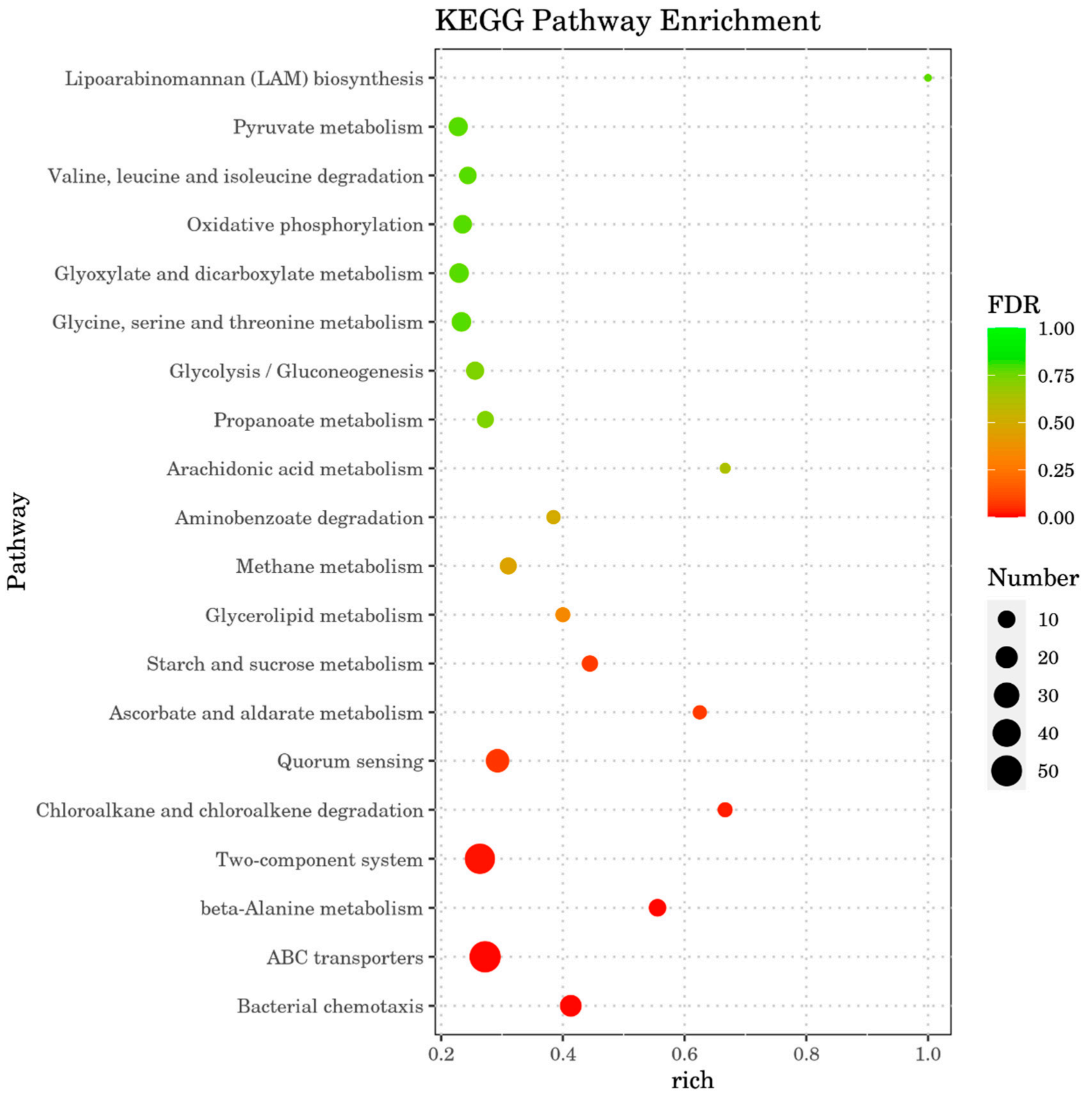

Figure S4 Comparison of growth conditions between salt-tolerant strain KT2440-*EcnhaA-betB* and wild-type strain KT2440 supplemented addition of proline and betaine with different concentrations of NaCl. Cell density measured at 48 h. The data are mean ± S.D. of three replicates.

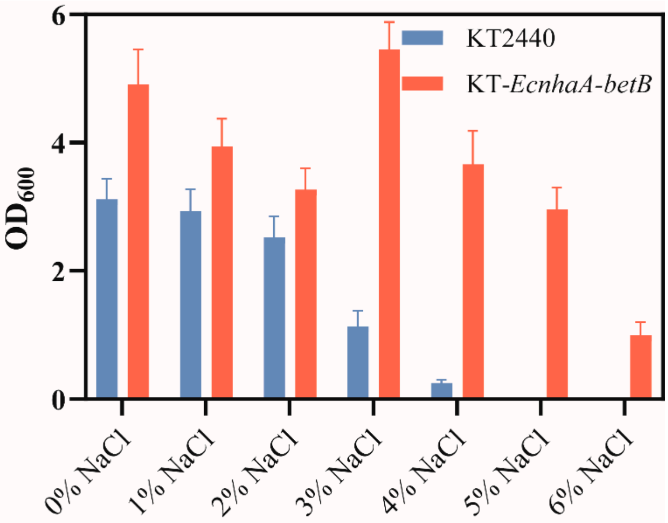

Table S1 Primers used in this study.

Primers	Sequence 5′→3′	Description
astA-F	ACAGGAAACAGAATTCATGATCGTTCGTCCTGTACGCA	Amplification of *astA*
astA-R	AGTCCGAGGCCTCGAGTTAATGGGCCTCCCGGCTG	
astC-F	ACAGGAAACAGAATTCATGTCCGTTGAGCAAGCCC	Amplification of *astC*
astC-R	AGTCCGAGGCCTCGAGTCAGCCTTTGGTCAGTGTGGC	
astD-F	ACAGGAAACAGAATTCATGACCACGCATTACATCGCAG	Amplification of *astD*
astD-R	AGTCCGAGGCCTCGAGTTATAGGGTGACGCCCGGC	
astE-F	ACAGGAAACAGAATTCATGCTCGCCCTTGGCAAAT	Amplification of *astE*
astE-R	AGTCCGAGGCCTCGAGTCACCCCAGGCGCTGC	
proI-F	ACAGGAAACAGAATTCATGAGCAAGACACGTATTGCCTT	Amplification of *proI*
proI-R	AGTCCGAGGCCTCGAGTTATTTGCCCAGTTGTTCGGCC	
proA-F	ACAGGAAACAGAATTCATGACTGAGTCCGTTCTTGACTATATGAC	Amplification of *proA*
proA-R	AGTCCGAGGCCTCGAGTCAGCAGGACCCCTGGC	
betA-F	ACAGGAAACAGAATTCTTGCACATGCCATCAGCCG	Amplification of *betA*
betA-R	AGTCCGAGGCCTCGAGTCAGCCCTGTTTCACTTCACGC	
betB-F	ACAGGAAACAGAATTCATGGCCCGTTTCGGAACG	Amplification of *betB*
betB-R	AGTCCGAGGCCTCGAGTCAGAAAACCGAGTTGTAGCCGC	
betT-III-F	ACACAGGAAACAGAATTCATGACTGCAGCCCCCAAACC	Amplification of *betT*-III
betT-III-R	AGTCCGAGGCCTCGAGTCAGCCCACCACGTGCAAGTAATG	
kdpA-F	ACACAGGAAACAGAATTCATGCACAGTTACGATTTCGCCTTGC	Amplification of *kdpA*
kdpA-R	AGTCCGAGGCCTCGAGGATCGGTCCTCAAAAACCCAAGCTAAGG	
kdpB-F	ACACAGGAAACAGAATTCATGAACATGCCCATTCCTGAAGTG	Amplification of *kdpB*
kdpB-R	AGTCCGAGGCCTCGAGTCAGACCAGGTTCAGGGCGTTG	
nhaA-II-F	ACAGGAAACAGAATTCGGAGCACCTGTGCCTATGCATAA	Amplification of *nhaA*-II
nhaA-II-R	AGTCCGAGGCCTCGAGAAGGGATCGCCAGTCCGGAATTG	
Ec*nhaA*-F	ACAGGAAACAGAATTCGTGAAACATCTGCATCGATTCTTTAGC	Amplification of Ec*nhaA*
Ec*nhaA*-R	AGTCCGAGGCCTCGAGTCAAACTGATGGACGCAAACGAAC	
He*ect*-F	ACACAGGAAACAGAATTATGAACGCAA CCACAGAGCC	Amplification of He*ectABC*
He*ect*-R	AGTCCGAGGCCTCGAGGCCTTCGATGCAATAAAC	
spuI-F	ACAGGAAACAGAATTCATGTCGGTACCCCCGCG	Amplification of *spuI*
spuI-R	AGTCCGAGGCCTCGAGTTACACGGTATGCAGGTACCAGTTG	
glnA-F	ACAGGAAACAGAATTCATGTCGAAGTCGGTTCAACTCATCAA	Amplification of *glnA*
glnA-R	AGTCCGAGGCCTCGAGTCAGCAGCTGTAGTACAGCTCGT	
dnaK-F	ACAGGAAACAGAATTCGCTGCTACCAAATTCAAGTTTCGG	Amplification of *dnaK*
dnaK-R	AGTCCGAGGCCTCGAGGCAGGGATTACTGCTTGTTGTTG	
dnaJ-F	ACAGGAAACAGAATTCGCAAATGACCTATGTCCAAGCGTG	Amplification of *dnaJ*
dnaJ-R	AGTCCGAGGCCTCGAGGTCGCATAACCTGTTCCTTGTCAG	
clpB-F	ACAGGAAACAGAATTCATGCGAATAGACCGTTTAACCAGC	Amplification of *clpB*

clpB-R	AGTCCGAGGCCTCGAGCTCATGGTGCAACACAATCAGCC	
htpG-F	ACAGGAAACAGAATTCTCGAAGACCATGACTGTGGAAACAC	Amplification of *htpG*
htpG-R	AGTCCGAGGCCTCGAGCCAGCGATTCGACGATTACTTTG	
betB-F1	CGTTTGCGTCCATCAGTTTGAATGGCCCGTTTCGGAACGCA	Amplification of *betB*
dnaJ-F1	CGTTTGCGTCCATCAGTTTGAATGACCTATGTCCAAGCGTG	Amplification of *dnaJ*
clpB-F1	CGTTTGCGTCCATCAGTTTGAATGCGAATAGACCGTTTAACCAGC	Amplification of *clpB*
dnaJ-F2	GCTACAACTCGGTTTTCTGAATGTCCAAGCGTGATTATTA	Amplification of *dnaJ*
clpB-F2	GCTACAACTCGGTTTTCTGAATGCGAATAGACCGTTTAAC	Amplification of *clpB*
clpB-F3	GCTTCTTCGGCGATCTCTGAATGCGAATAGACCGTTTAAC	Amplification of *clpB*
16s-F	CGACAGAATAAGCACCGGCTAACTC	Primers used for qRT-PCR
16s-R	CTCTACCGTACTCTAGCTTGCCAG	
nhaA-II-1	GTTCAGCCTACGCCTATGTCATTCC	
nhaA-II-2	CATTGCGGTGACAGGTGTCATCAG	
kdpA-1	TCCAGTGGCATTGGTGTTCACCTTC	
kdpA-2	GTCTGTTGCACCTGCGTGCTTTCCA	
kdpB-1	GATTGTCGAATACCTGCGCCAGTTG	
kdpB-2	CATTTCCAGGCGTTGCATACCGAC	
betT-III-1	CGTGGGTTTGTTCATCGCACGTATTTC	
betT-III-2	GCTCCAGGAAAGCAAACAACGCGA	
betA-1	GACATCCCGGTTGTGCACGA	Primers used for qRT-PCR
betA-2	CATTGAACATCCACTCGGCACC	
betB-1	TCGCCATGATGGCCAACTTCTACAG	
betB-2	TTCCATGTGCTGGAAGCTGACCAG	
astA-1	AGCCTGCTGACCAGTTTCTATGTG	
astA-2	CATAGTTGAGGTCGAAGAAGTTGCGG	
astB-1	CGTTTGGGCAAGTGTAATGTGCAAC	
astB-2	CTCCTGACAAGATCGGCTTGCAA	
astC-1	CAAGCTGTGTGACGAGCACAAC	
astC-2	CTCTTGGCACTGGTCAGAATGTCC	
astD-1	GGTGTATACCATTATCCAGTCGGCG	
astD-2	CAGCGAAATCACCGAGCCCATGAAA	
astE-1	GAAGATCGAGCAATTCGCCTTGTAC	
astE-2	GCTTCAGCCTCAAGTTGCTCGTAG	
proA-1	TGCCGGTGATCAAGCACCTG	
proA-2	GTCATGGCCTCGATCAGCAGGAAG	
proI-1	TCTGCAGGCCGAACAACTGCTGTC	
proI-2	GTCATGGCCTCGATCAGCAGGAAG	
glnA-1	CGTTGAAGTTCACCACCACGAAG	
glnA-2	GTACAGTGGCTTCGGCATGAAG	
glgA-1	CTGGCGTACCAAGGTGTGTACAG	
glgA-2	CTGACTGTGGTGATGTGGCTGGA	
glgB-1	GTATCGGTGTGCTGCTGGACTG	
glgB-2	GTAGATCAGCGTGTTCCAGTCCTG	

glgX-1	CTCAGCCACGAACTGGATGAAGTG	
glgB-2	CTCATGCGCTCGATCTGGCTTTG	
treSB-1	ACAACTTCACCGCTTCGCTGTAC	
treSB-2	GACTTGTAGCCGTTGAAGTCCAGG	
treZ-1	GTGCGCGAGTACTTTATCCAGAATG	
treZ-2	CAACTCGATCAGGAAGTCGGGC	
treY-1	TTCTTCCAGCAGGCCCTGACCAATG	
treY-2	CAGTCAGTTGCTGGAAGCGCAC	

Table S3 Sequence of He*ect* cluster. The synthetic He*ect* cluster was provided by GenScript Biotech Corporation. Black represents He*ectA*, red represents He*ectB* and green represents He*ectC*. Underline indicates the RBS sequence.

ATGAACGCAACCACAGAGCCCTTTACACCCTCCGCCGACCTGGCCAAGCCCAGCGTGGCCGATGCCG
TGGTCGGCCATGAGGCCTCACCGCTCTTCATCCGCAAGCCAAGCCCCGATGACGGCTGGGGCATCTAC
GAGCTGGTCAAGTCCTGTCCGCCTCTCGACGTCAATTCCGCCTACGCCTATCTGTTGCTGGCCACCCAG
TTCCGCGATAGCTGCGCCGTGGCGACCAACGAAGAGGGCGAGATCGTCGGCTTCGTTTCCGGCTACGT
GAAGAGCAACGCCCCCGATACCTATTTCCTCTGGCAGGTTGCCGTGGGCGAGAAGGCACGTGGCACC
GGCCTGGCCCGTCGTCTGGTGGAAGCCGTGATGACACGCCCGGAAATGGCCGAGGTCCACCATCTCG
AGACCACTATCACGCCCGACAACCAGGCGTCCTGGGGCTTGTTCCGCCGTCTCGCCGATCGCTGGCAG
GCGCCGTTGAACAGCCGCGAATACTTCTCCACCGATCAACTCGGCGGTGAGCATGACCCGGAAAACC
TCGTTCGCATCGGCCCGTTCCAGACCGACCAGATCTGAAGGAGGAATATACCGGTACCATGCAGACCC
AGATTCTCGAACGCATGGAGTCCGACGTTCGGACCTACTCCCGCTCCTTCCCGGTCGTCTTCACCAAG
GCGCGCAATGCCCGCCTGACCGACGAGGAAGGGCGCGAGTACATCGACTTCCTGGCCGGTGCCGGCA
CCCTGAACTACGGCCACAACAACCCGCACCTCAAGCAGGCGCTGCTCGACTATATCGACAGCGACGG
CATCGTCCACGGCCTGGACTTCTGGACTGCGGCCAAGCGCGACTATCTGGAAACCCTGGAAGAGGTG
ATCCTCAAGCCGCGCGGTCTCGACTACAAGGTGCATCTGCCCGGACCGACTGGCACCAACGCCGTCG
AGGCGGCCATTCGCCTGGCCCGGGTCGCCAAGGGGCGCCACAATATCGTCTCCTTCACCAACGGCTTT
CATGGCGTCACCATGGGCGCGCTGGCGACCACCGGTAACCGCAAGTTCCGCGAGGCCACCGGTGGCG
TGCCGACCCAGGCTGCTTCCTTCATGCCGTTCGATGGCTACCTCGGCAGCAGCACCGACACCCTCGAC
TACTTCGAGAAGCTGCTCGGCGACAAGTCCGGCGGCCTGGACGTGCCCGCGGCGGTGATCGTCGAGA
CAGTGCAGGGCGAGGGCGGTATCAATGTCGCCGGCCTGGAGTGGCTCAAGCGCCTCGAGAGCATCTG
CCGCGCCAATGACATCCTGCTGATCATCGACGACATCCAGGCGGGCTGCGGCCGGACCGGCAAGTTCT
TCAGCTTCGAGCATGCCGGCATCACGCCGGATATCGTGACCAACTCCAAGTCGCTGTCCGGTTACGGC
CTGCCGTTCGCTCACGTCCTGATGCGCCCCGAGCTCGACAAGTGGAAGCCCGGTCAGTACAACGGCA
CCTTCCGCGGCTTCAACCTGGCTTTCGCCACTGCTGCTGCCGCCATGCGCAAGTACTGGAGCGACGAC
ACCTTCGAGCGTGACGTGCAGCGCAAGGCTCGCATCGTCGAGGAACGCTTCGGCAAGATCGCCGCCT
GGCTGAGCGAGAACGGCATCGAGGCCTCCGAGCGCGGCCGCGGGCTGATGCGGGGCATCGACGTGG
GTTCCGGCGATATCGCCGACAAGATCACCCACCAAGCCTTCGAGAACGGGTTGATCATCGAAACCAGC
GGTCAGGACGGCGAAGTGGTCAAGTGCCTGTGCCCGCTGACCATTCCCGACGAAGACCTGGTCGAGG
GACTCGACATCCTCGAGACCAGCACCAAGCAGGCCTTTAGCTGAAGGAGGAATATACCGGTACCATGA

TCGTTCGCAATCTCGAAGAAGCGCGCCAGACCGACCGTCTGGTCACCGCCGAAAACGGCAACTGGGA

CAGCACCCGCCTGTCGCTGGCCGAAGATGGTGGCAACTGCTCCTTCCACATCACCCGCATCTTCGAGG

GTACCGAGACCCACATCCACTATAAGCATCACTTCGAGGCTGTTTATTGCATCGAAGGC
