# Supplementary figures and images for: Improvement in Salt Tolerance Ability of Pseudomonas putida KT2440
Source: Biology (Basel). 2024 Jun 1;13(6):404. doi: 10.3390/biology13060404 (PMC11200750; doi:10.3390/biology13060404)

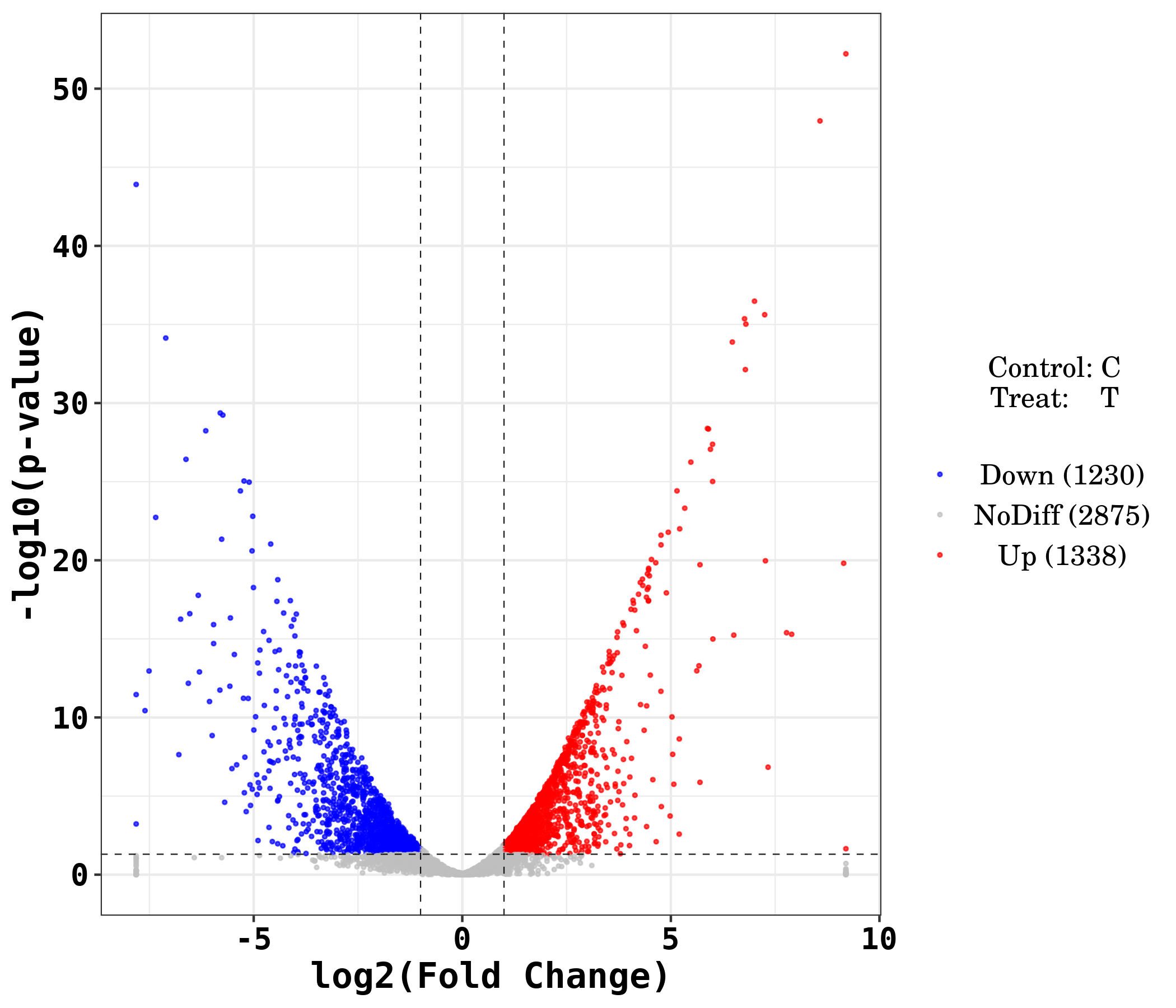

Supplement: Supplementary file 1 [file biology-13-00404-s001.zip › Supplemental Material/Figure S1.tif]

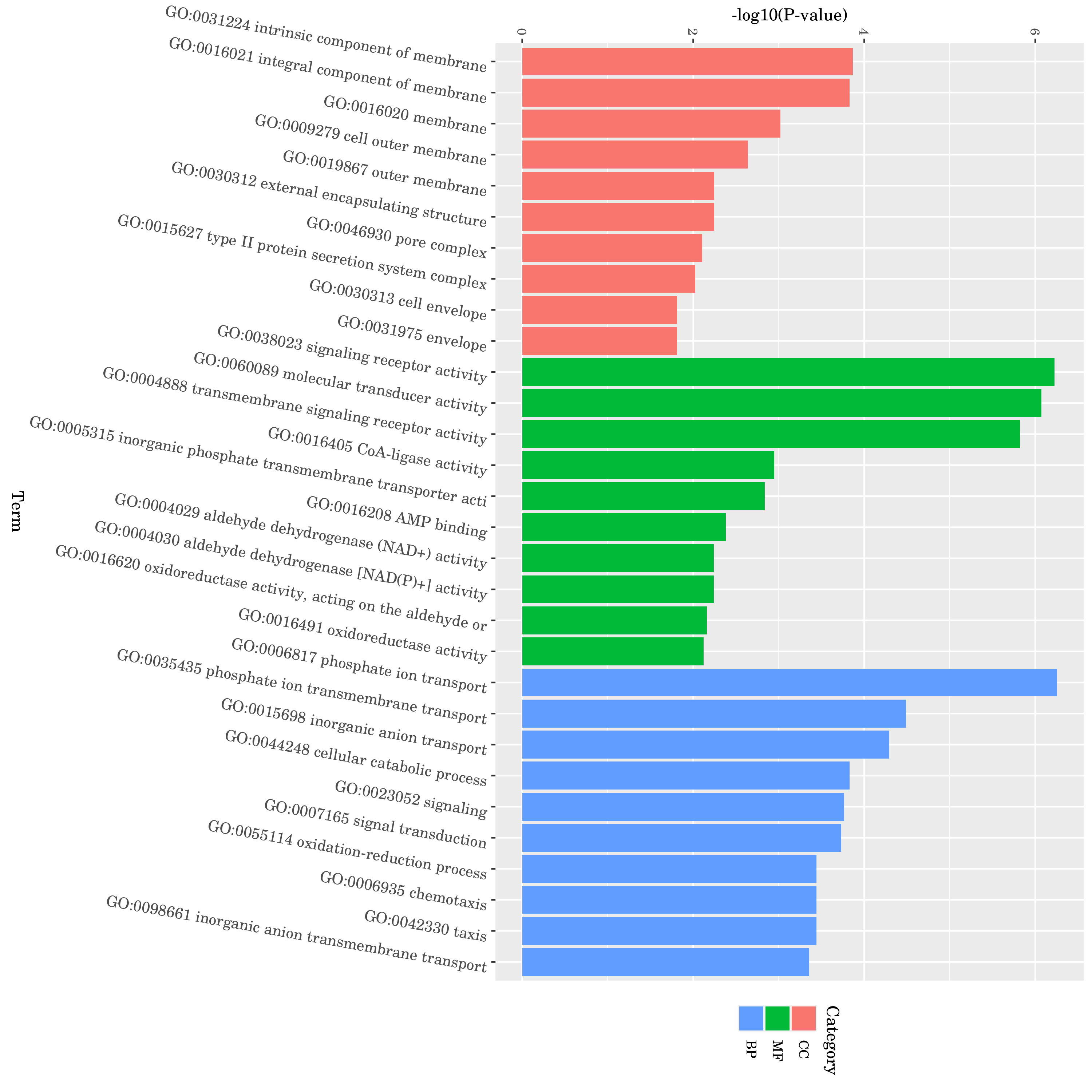

Supplement: Supplementary file 1 [file biology-13-00404-s001.zip › Supplemental Material/Figure S2.tif]

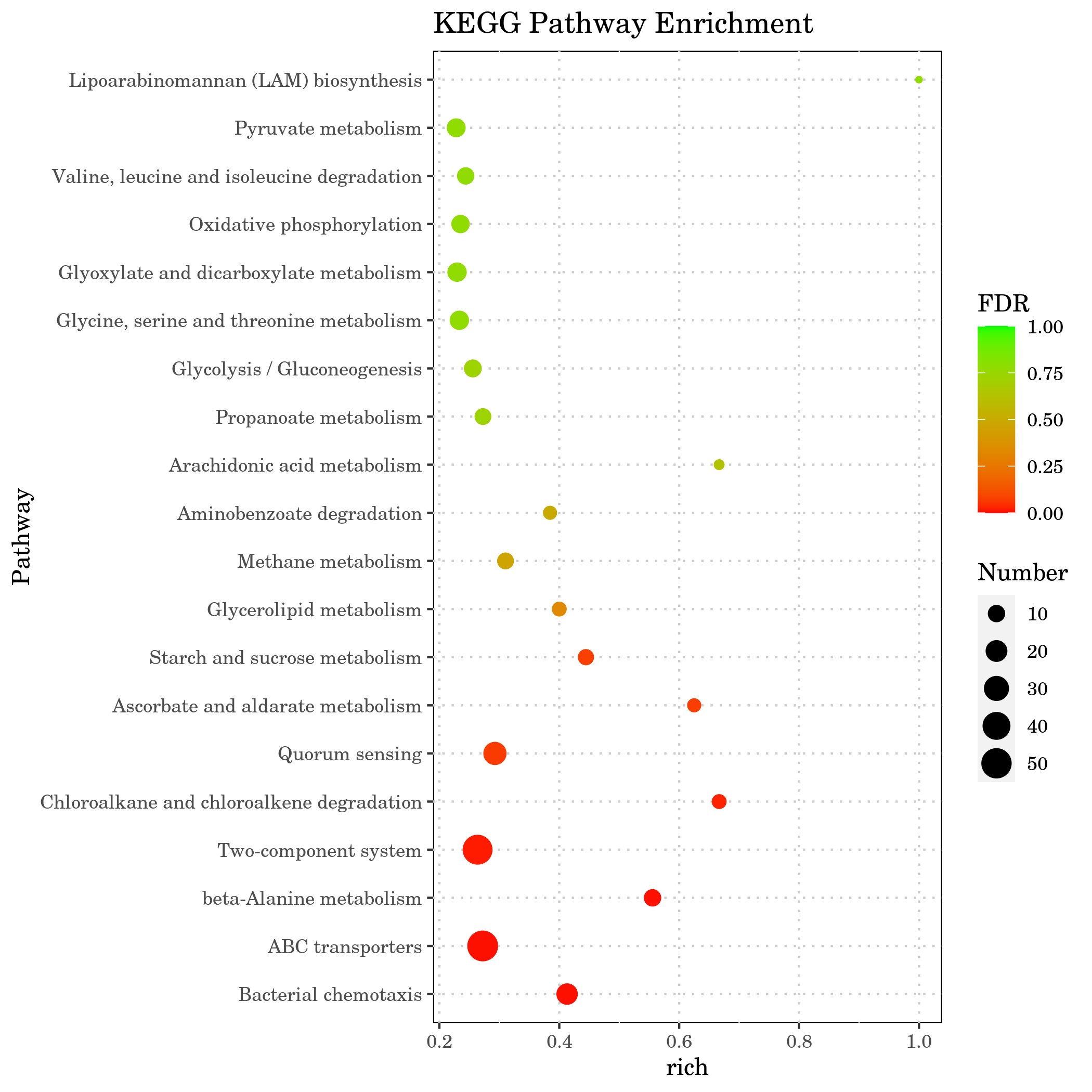

Supplement: Supplementary file 1 [file biology-13-00404-s001.zip › Supplemental Material/Figure S3.tif]

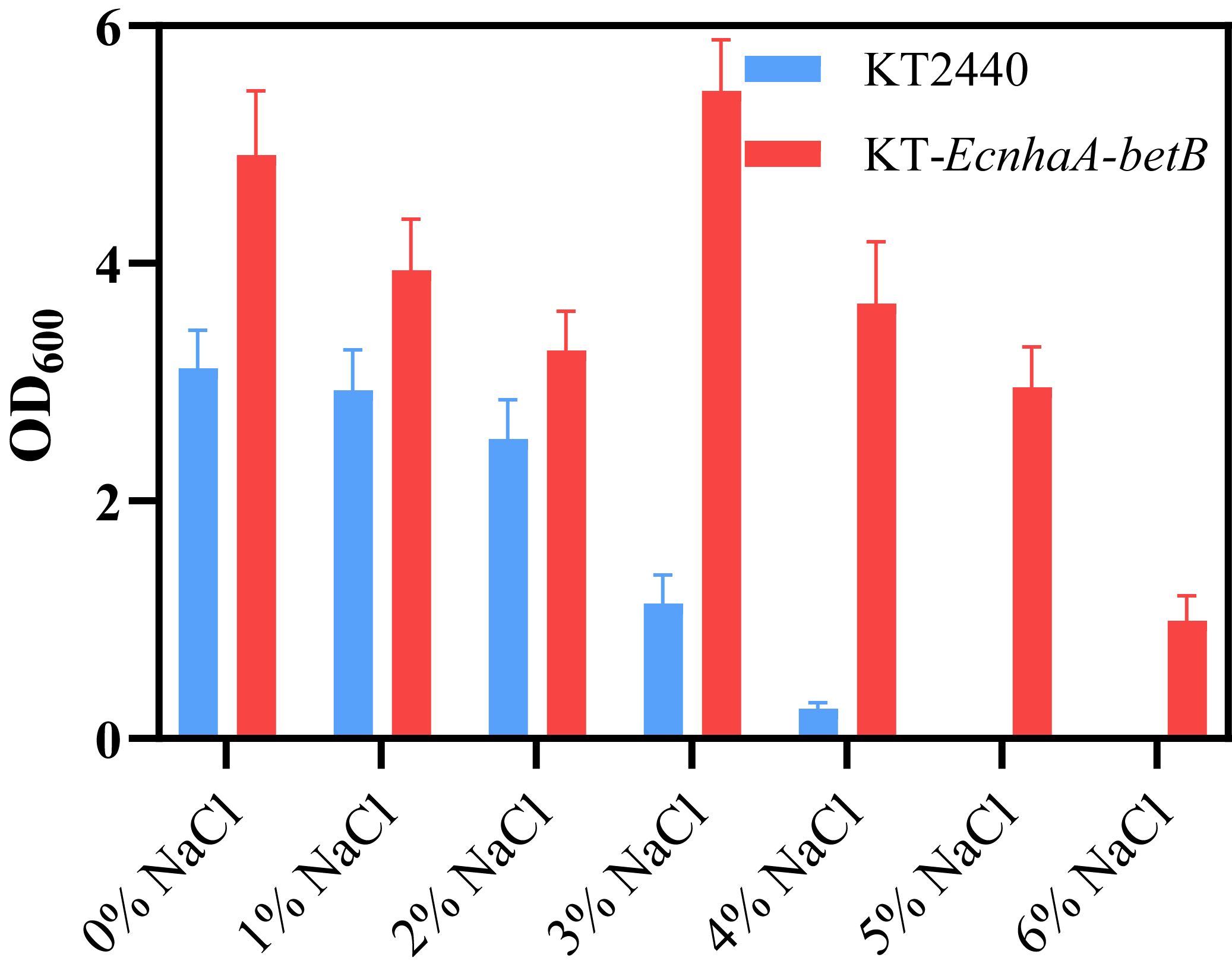

Supplement: Supplementary file 1 [file biology-13-00404-s001.zip › Supplemental Material/Figure S4.tif]
